# Normative data for three tests of visuocognitive function in primary school children: cross-sectional study

**DOI:** 10.1136/bjophthalmol-2014-305868

**Published:** 2015-02-23

**Authors:** Cathy Williams, Iain D Gilchrist, Sue Fraser, HM McCarthy, Julie Parker, Penny Warnes, Jill Young, Lea Hyvarinen

**Affiliations:** 1 School of Social and Community Medicine, University of Bristol, Bristol, Avon, UK; 2 Bristol Eye Hospital, University Hospitals of Bristol Foundation NHS Trust, Bristol, Avon, UK; 3 School of Experimental Psychology, University of Bristol, Bristol, Avon, UK; 4 Emerson's Green Primary School, Bristol City Council, Bristol, Avon, UK; 5 Rehabilitation Sciences, TU Dortmund University, Dortmund, Germany.

**Keywords:** Visual perception, Child health (paediatrics), Epidemiology, Rehabilitation, Diagnostic tests/Investigation

## Abstract

**Background/Aims:**

There is an increasing recognition that visuocognitive difficulties occur in children with neurodevelopmental problems. We obtained normative data for the performance of primary school children using three tests of visuocognitive function that are practicable in a clinical setting.

**Methods:**

We tested 214 children aged between 4 and 11 years without known developmental problems, using tests to assess (1) orientation recognition and adaptive movement (postbox task), (2) object recognition (rectangles task) and (3) spatial integration (contours task).

**Results:**

96% could do the postbox task with ease—only 4% (all aged <9 years) exhibited minor difficulties. Errors in the rectangles task decreased with age: 33% of children aged 4–5 years had major difficulties but >99% of children aged ≥6 years had no, or minor, difficulties. Median scores for the contours task improved with age, and after age 8 years, 99% could see the contour using long-range spatial integration rather than density.

**Conclusions:**

These different aspects of children’s visuocognitive performance were testable in a field setting. The data provide a benchmark by which to judge performance of children with neurodevelopmental problems and may be useful in assessment with a view to providing effective supportive strategies for children whose visuocognitive skills are lower than the expectation for their age.

## Introduction

Cognitive functions that relate to vision (visuocognitive abilities) have a developmental profile just like other cognitive processes such as language acquisition.^1^ Two primary cortical networks mediate visual information: the dorsal stream, which is related to the generation of visually guided actions, and the ventral stream, which is related to the recognition of objects and pictures.^2^

Visuocognitive abilities can be selectively impaired in children with neurogenetic disorders, such as Williams syndrome or Down syndrome,^3^ or after early acquired neuronal damage as in prematurity^4^ and cerebral palsy.^5^ Visuocognitive problems often coexist with optic nerve disorders, ocular or refractive impairments.^5^ ^6^ Many exist for visuocognitive functions and some have been adapted for children in research settings.^7^ ^8^ Available clinical tools include a question inventory^9^ ^10^ and a test battery for children aged up to 4 years;^11^ however, additional direct assessments for school-aged children are needed.

We wished to obtain normative data from primary school-aged children, for three tests that would be easy to administer in a clinical setting. Two of the tests (postbox task and rectangles task) are available commercially on a specialist vision-testing website (http://www.lea-test.fi/) and the third test (contour task) had been made available to us as part of a previous research study and is used as a test of visual processing that matures during childhood and may be impaired in the presence of neurodevelopmental conditions such as migraine.^12^

The postbox task involves perceiving orientation and then adjusting hand movement accordingly, in the form of posting a ‘letter’ through a ‘letter-box’. Goodale *et al*^13^ used this task to demonstrate that the neural substrates responsible for perceiving orientation of a slot were different from those responsible for programming accurate hand orientation to the slot. The task was later adapted for young children and made commercially available, with the rectangles task, in 1996 (http://www.lea-test.fi). Similar paediatric modification was used to demonstrate that children with Williams syndrome performed differently from typically developing children.^14^

The rectangles task is a modification of a classical test of visual perception called the Efron test, in which a person has to match rectangles of the same total surface area but varying proportions.^15^ Difficulty with this task is classically linked to difficulty in *reproducing* visual objects (apperceptive agnosia), as opposed to difficulty in *recognising* visual objects (associative agnosia),^16^ and this distinction has been described in some patients, for example, a 13-year-old child who had undergone removal of her dysplastic right occipital lobe at the age of 7 years because of intractable epilepsy and on later testing at the age of 13 years was diagnosed with visual agnosia and autism.^17^

The contour task assesses the ability to integrate information across areas of the visual field and is assumed to rely on neurons that support long-range facilitation.^18^ This ability improves during childhood and adolescence^19^ ^20^ and is abnormal in children with disrupted visual development due to amblyopia or strabismus.^21^

We aimed to collect normative data using these three visual tasks in a primary school.

## Methods

We obtained approval from the University Faculty of Science Ethical Committee and approval from the Head teachers of each of two mainstream schools. We sent letters explaining the study and consent forms, then visited the school and saw all children whose parents had returned a signed consent form.

The school provided details of name (which was not stored), date of birth and any conditions known to the teachers that affected the children's development or education. This information was not available to the testers until after the testing sessions.

### Protocol for testing

All children were tested according to a predesigned protocol. Binocular visual acuity (at 4 m) was tested with a Keeler logarithm of the minimum angle of resolution (LogMAR) crowded test (Keeler) or crowded Kay Pictures (Kay Pictures), with glasses if worn. Next, the children were taken to a nearby room and were asked to carry out the three tasks: contour task, the postbox task and then the rectangles test. Exact timings were not kept but total testing for each child took approximately 10 min.

### Contour task procedure

**Figure 1:**
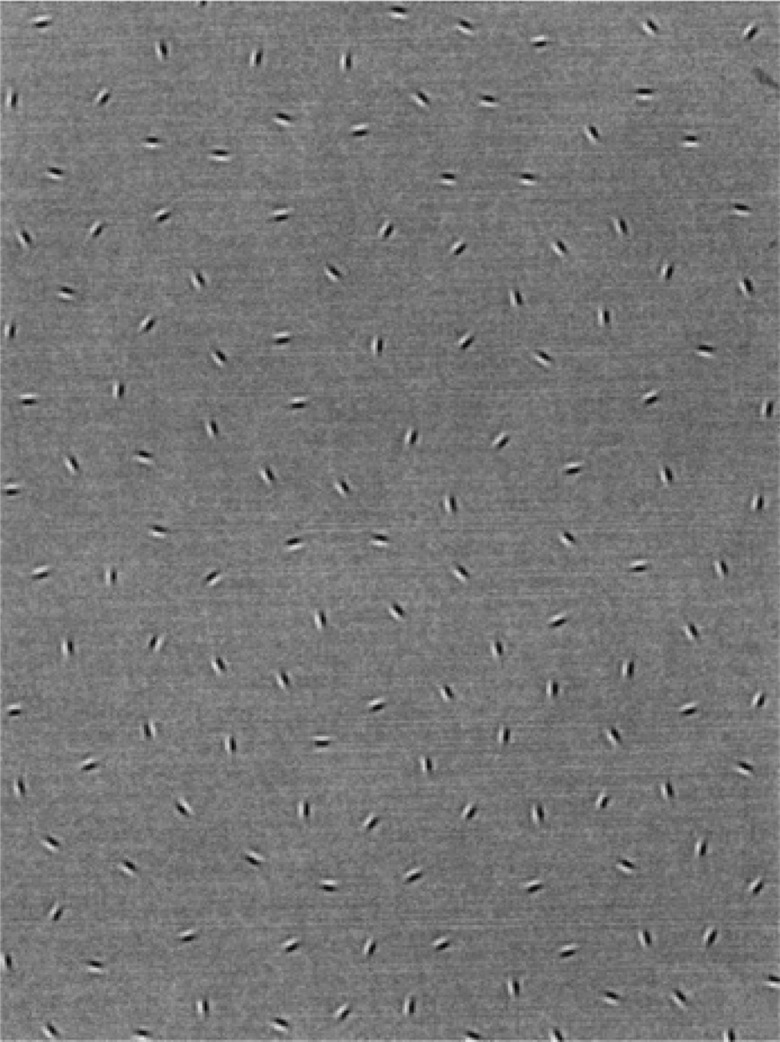
The contour test card. Picture of the demonstration test plate with the closed contour ‘potato’ shape.

The outcome measure for this test is Δ, which is the ratio of the spacing between the contour-defining elements (Gabor patches, which are oval black-and-white-striped shapes) and the spacing between the randomly arranged background-element Gabor patches (see figure 1). First, the children were shown a demonstration card and were encouraged to use their fingers to outline the ‘potato’ shape. Then they were shown subsequent cards where the ‘Δ’ decreased in 0.05 steps. A staircase procedure was used, turning the cards 90° for repeat presentations, so the final score was the card with the smallest Δ where the ‘potato’ shape was correctly identified twice and the subsequent card was not identified twice.^21^ Testing was carried out binocularly, with glasses if worn.

### Grading for the postbox and rectangles tasks

For the postbox and rectangles tasks we devised a simple semiquantitative system to describe each child's performance: 1=no problems; 2=minor/moderate difficulties and 3=major difficulties or could not do the task.

### The postbox task procedure

After demonstrating they could pronate and supinate their wrists, the children were given a plastic card approximately 10 cm×10 cm and were shown a blue circular disc (diameter 20 cm) with a central slit approximately 3 cm×15 cm. They were asked to ‘post’ the card through the slit. First four presentations were with the card in the same orientation as the slit (horizontal, vertical, oblique×2) and then four presentations involved the card being at a 90° angle to the slit.

**Figure 2:**
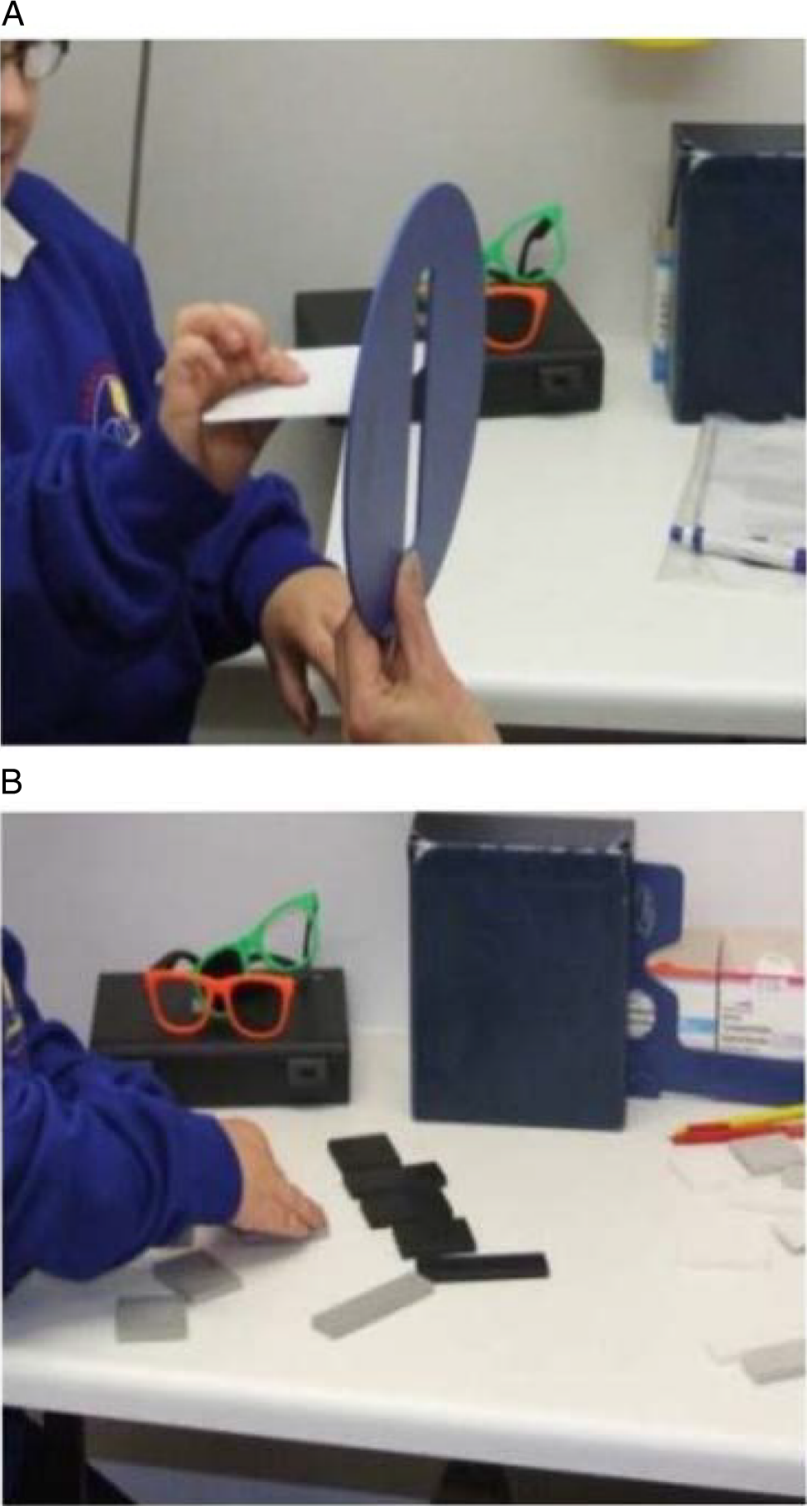
(A) The postbox task and (B) the rectangles task.

The postbox task is illustrated in figure 2A.

### The rectangles task procedure

We designed a novel protocol for this task as we hypothesised that for some children it may be harder to copy the spatial arrangement of scattered shapes than to copy one more complex shape. Children were initially shown two similarly coloured rectangles, a long thin one and a shorter broader one, and were asked to identify which was which.

The examiner then asked the children to close their eyes and placed five similarly coloured, but variously shaped rectangles, in a standard pattern on the table. The child had a similar set of rectangles, but in a different colour, randomly displayed in front of them and was asked to copy the examiner's rectangles. This was done twice, once with a ‘closed’ pattern—all the rectangles touching in the examiner's pattern and again with an ‘open’ pattern where the examiner's rectangles were spaced apart by approximately 1–2 cm.

The examiners noted the accuracy of the child's reproduction of the examiner's pattern using the scale 1–3 and also whether the child used tactile information, for example, putting rectangles on top of each other to confirm that the sizes were equal. The rectangles test is illustrated in figure 2B.

### Validation study

Some children were video recorded when doing the postbox and rectangles tasks. The videos were then graded using the same scoring scheme independently by two examiners.

### Results

We examined 231 children of whom 17 were reported to have a condition affecting development/education: four had Down syndrome, five had cerebral palsy, two had visual impairment, two had colour deficiency, one had cerebral visual impairment and three for no details were given. The results from these children are not presented. Of the 214 children not reported to have any conditions, 103 (48%) were boys and the ages are shown in table 1.

**Table 1:** Age of children participating in study

Age group (years)	Children (n)
<5	15
5–6	26
6–7	42
7–8	47
8–9	40
9–10	22
10–11	22
Total	214

**Figure 3:**
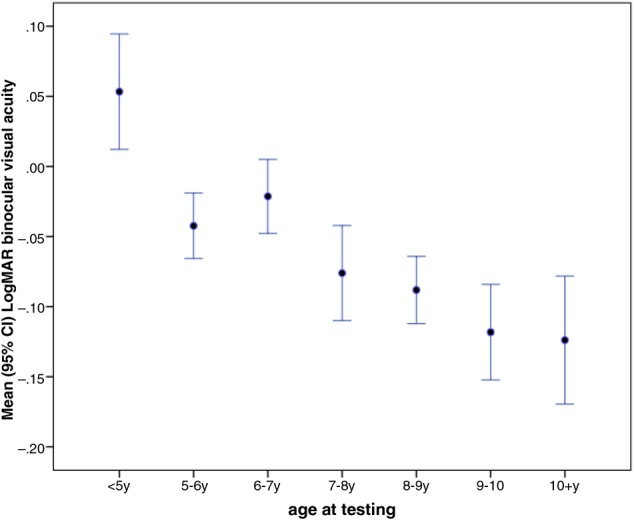
Mean binocular logarithm of the minimum angle of resolution (LogMAR) visual acuity (95% CI) by age for 214 children without known neurodevelopmental problems.

The mean binocular visual acuity results are shown in figure 3. Ninety nine per cent (211/213) had binocular acuity of at least 0.2 LogMAR (approximately 6/9 on a Snellen chart) and two children (one aged 6–7 years and one aged 7–8 years) had binocular acuity of 0.3 and 0.4, respectively, and for one the data were missing.

### Repeatability of assessment

Nineteen children without known problems were graded independently by two examiners. No child was graded ‘3’ on any task by either grader. Agreement between the graders is summarised in table 2 (κ, SE) and would be described as ‘fair’ for the postbox and either ‘fair’ or ‘poor’ for the two rectangles tasks.^22^

**Table 2:** Repeatability of the postbox and rectangles tasks

Task	Grader 1 Children graded as ‘1’	Grader 1 Children graded as ‘2’	Grader 2 Children graded as ‘1’	Grader 2 Children graded as ‘2’	κ (SE)
Postbox	19	1	15	5	0.27 (0.22)
Rectangles open	16	3	14	5	0.38 (0.25)
Rectangles closed	15	3	14	4	0.12 (0.26)

## Results

### Contour task

Two children could do the test plate only: both were aged under 7 years.

The mean, SD, median and 5th and 95th centile Δ scores for the remaining 212 children are shown in table 3. The mean scores gradually increased with age. The median scores were stable at 0.95 across most of the age range but the 5th–95th centile ranges gradually decreased (performance improved). At least 95% of children aged ≥8 years could see the card where the spacing of the distracters was equal to that of the contour (Δ=1.0), where target is detected by long-range integration of the Gabor patch orientation.

**Table 3:** Mean (SD), median, 5th centile and 95th centile Δ scores for the contour test by age category in 215 children with no known developmental problems

Age (years)	n	Mean	SD	Median Δ	5th centile	95th centile
<5	13	1.03	0.09	1.0	1.20	0.95
5 to <6	26	1.00	0.09	0.95	1.20	0.92
6 to <7	41	0.95	0.08	0.95	1.10	0.76
7 to <8	47	0.93	0.05	0.95	1.03	0.82
8 to <9	40	0.93	0.06	0.95	1.00	0.85
9 to <10	22	0.87	0.12	0.95	0.95	0.57
≥10	22	0.92	0.07	0.95	1.00	0.72
Total	212	0.95	0.09	0.95	0.84	1.10

The data for two children (one aged <5 years, another aged 6–7 years) were missing for the contour task.

### Postbox test

Data were missing for 7/214 children. Across all ages, 199/207 (96%) were scored ‘1’ (no difficulty). Of the 8/207 (4%) children who were noted as having mild/moderate difficulty, four were inaccurate (ie, needed repositioning) with the oblique presentations and four banged the letter across the slit, one constantly and three only when slit and letter were at different orientations. Six of these children were aged ≤6 years.

### Rectangles task

All 214 children could correctly identify a tall rectangle from a short, and their scores are shown in table 4. In both conditions, the children's performance improved with age and at least 95% of children could do the task without major difficulties from the age of 6 years onwards. However, 9% of even the oldest children displayed minor/moderate difficulties.

**Table 4:** Examiners’ scores of 214 children performing the rectangle-matching tests with either closed or open spacing

Age (years)	n	Closed			Open		
		Major difficulties N (%)	Minor difficulties N (%)	No difficulties N (%)	Major difficulties N (%)	Minor difficulties N (%)	No difficulties N (%)
<5	15	5 (33.3)	5 (33.3)	5 (33.3)	4 (26.7)	8 (53.3)	3 (20.0)
5–6	26	3 (11.5)	9 (34.6)	14 (53.8)	4 (15.4)	5 (19.2)	17 (65.4)
6–7	42	0 (0)	11 (26.2)	31 (73.8)	2 (4.8)	10 (23.8)	30 (71.4)
7–8	47	1 (2.0)	10 (20.4)	36 (77.6)	2 (4.3)	6 (12.8)	39 (83.0)
8–9	40	0 (0)	6 (14.6)	34 (85.4)	1 (2.5)	6 (14.6)	33 (82.9)
9–10	22	0 (0)	4 (18.2)	18 (81.8)	0 (0)	5 (22.7)	17 (77.3)
11+	22	0 (0)	2 (9.1)	20 (90.9)	0 (0)	2 (9.1)	20 (90.9)
Total	214	9 (4.2)	47 (22.0)	158 (73.8)	13 (6.1)	42 (19.6)	159 (74.3)

Agreement between the two task conditions was moderate: κ (SE) was 0.47 (0.06). Of the 214 children, 139 (65%) children managed both with ease, 6 children (3%) had major difficulties with both conditions and the remaining 69 children (32%) were evenly spread between those who had minor difficulties both times (n=23) or were better at the closed condition (n=23) or the open condition (n=23).

Only 11/214 (5.1%) of the children used touch to help match the rectangles. All were aged ≤9 years.

### Performance across all tests

There were 7/214 (3.3%) for whom there was no score written for the postbox result and of these none had major problems with the rectangles and all did the contour task.

The majority of the 207 children with data for all three tasks (postbox and both rectangles presentations) performed them all easily: 133/207 (64.3%) scored ‘1’ for all three tasks, a further 53/207 (25.6%) had no errors with the postbox and only minor/moderate errors with the rectangles; 13/207 (6.3%) had no difficulties with the postbox but major problems with the rectangles and 8/207 (3.9%) had minor difficulties with postbox of whom three also had major difficulties with the rectangles. Therefore, across all ages 191/207 (92.3%) children could do the postbox and both rectangles tests without major difficulties.

The results of the contour task varied with age. Of the 5/212 (2.4%) children with data who got the lowest score possible (Δ of 1.2), two of them also had major difficulties with both the rectangles task presentations and both children were under the age of 6 years.

In summary, few children had major difficulties with any task and those who did were either in the youngest age groups and/or had difficulties with more than one test.

## Discussion

We were able to administer three visuocognitive tests to primary school children. The repeatability of the grading from 1 to 3 of the child's performance was only moderate but there was a clear decrease in numbers of children observed to have difficulties with increasing age.

Over 95% of the children could do the postbox task easily, whereas our modification of the rectangles task was harder for the younger children and it was not until they were ≥6 years that at least 95% could do the task without major problems. We observed minor/moderate errors in the performance of 9% of even the oldest children. This may be a reflection of the only moderate agreement between testers and/or may indicate that performance in this test is not maximal by the age of 11 years. The scores for the contour task improved with age as previously reported.^23^

Our study has several limitations. The number of children sampled was small, only two schools took part and the validation study was small. We did not store details of ethnicity but the population resident around each school is predominantly Caucasian and English speaking—our results may not generalise to children from other social backgrounds. We did not assess the children formally for their cognitive, motor or developmental skills so we have misclassified some children with undiagnosed problems as ‘normal’, thus our age-specific ranges for expected performance may be wider than would be obtained if we had been able to exclude any previously unknown problems. We did not test monocular vision or stereopsis or exclude children with strabismus which may have lowered our observed norms for the contour task that can be abnormal in the presence of these conditions.^24^ We designed a new procedure for the rectangles task, which may give different results from those obtained if using the original set of instructions (http://www.lea-test.fi/index.html?start=en/vistests/instruct/pvrectan/pvrectan.html).

The advantage of our approach is that we were able to assess over 200 primary school children in only a few minutes and in a naturalistic setting. This is likely to mean similar testing may be feasible in eye clinics or special schools elsewhere in the UK and possibly in other countries. There is a conflict between increasing recognition of visuocognitive dysfunctions in children and the limitations of time and resource within health services. This suggests a need for robust clinical assessments of visuocognitive functions that can help bridge the gap between either not addressing these functions at all or having detailed 1–2 h neuropsychological assessments that only few children can access. A knowledge of normal development is important to avoid describing behaviour or test results as ‘abnormal’ when, in fact, they are representative of what is normal at a particular age. In summary, therefore, we present normative data on three tests suitable for use in clinical settings with primary school-aged children, as a guide to what is age appropriate with these tests. The data may be useful when assessing visuocognitive functions in children with known or suspected neurodevelopmental problems.
